# Humidity Sensitivity of Multi-Walled Carbon Nanotube Networks Deposited by Dielectrophoresis

**DOI:** 10.3390/s90301714

**Published:** 2009-03-11

**Authors:** Litao Liu, Xiongying Ye, Kang Wu, Rui Han, Zhaoying Zhou, Tianhong Cui

**Affiliations:** 1 The state Key Laboratory of Precision Measurement Technology and Instruments, Department of Precision Instruments, Tsinghua University, Beijing 100084, P.R. China; E-mails: liulitao00@mails.tsinghua.edu.cn (L.T. Liu); wuk05@mails.tsinghua.edu.cn (K. Wu); gdla2008@yahoo.com.cn (R. Han.) zhouzy@mail.tsinghua.edu.cn (Z.Y. Zhou);; 2 Department of Mechanical Engineering, University of Minnesota, Minneapolis, Minnesota, MN 55455, USA; E-Mail: tcui@me.umn.edu (T. Cui)

**Keywords:** Humidity sensitivity, Multi-walled carbon nanotubes, Dielectrophoresis, Thermal annealing

## Abstract

This paper presents an investigation on the humidity sensitivity of deposited multi-walled carbon nanotube (MWCNT) networks using ac dielectrophoresis (DEP) between interdigitated electrodes (IDEs). MWCNTs dispersed in ethanol were trapped and enriched between IDEs on a Si/SiO_2_ substrate under a positive DEP force. After the DEP process, the ethanol was evaporated and the MWCNT network on a substrate with IDEs was put into a furnace for repeated thermal annealing. It was found that the resistance stability of the network was effectively improved through thermal annealing. The humidity sensitivity was obtained by measuring the resistance of the MWCNT network with different relative humidity at room temperature. The experimental results show the resistance increases linearly with increasing the relative humidity from 25% to 95% RH with a sensitivity of 0.5%/%RH. The MWCNT networks have a reversible humidity sensing capacity with response time and recovery time of about 3 s and 25 s, respectively. The resistance is dependent on temperature with a negative coefficient of about −0.33%/K in a temperature range from 293 K to 393 K.

## 1. Introduction

Carbon nanotubes (CNTs) are well-known functional materials with unique electrical, physical, mechanical, and chemical properties. A variety of promising applications have been considered, including sensors, field emission displays, nanoelectronic devices, conductive composites, etc [1]. There have been high expectations for CNTs as novel sensing materials, since their hollow cores with a large surface area to volume ratio are well suited for physical adsorption or chemical interaction with sensed targets. As a result, CNT-based gas sensors [2–5] have received much attention because of their outstanding properties such as faster response, higher sensitivity, and lower operating temperature. Recently, some reports showed that CNT films could be also used as a humidity sensing material due to a drastic change in the electrical conductivity or capacitance upon the adsorption of water molecules. There are mainly two methods to form CNT films for humidity sensors. One is to directly grow CNTs on electrodes by chemical vapour deposition (CVD) [6,7]. The other is to drop-cast a CNT suspension or a CNT composite on a substrate with electrodes [8,9]. However, the CNTs in these films have a high density and/or are random. Their sensitivities are usually not linear, and their response and recovery times are relatively long. Dielectrophoresis (DEP) is an effective method to deposit and align CNTs on electrodes. The advantages of DEP are that the number of CNTs trapped in the desirable region can be well controlled and that all of CNTs are almost regularly aligned across the electrodes.

In this paper, we report an investigation on humidity sensitivity of deposited MWCNT networks using ac DEP between interdigitated electrodes (IDEs). Thermal annealing was used to improve the resistance stability of the network. Humidity sensitivity of the MWCNT network was investigated at room temperature, and the effect of the temperature on resistance changes of the MWCNT network was also studied.

## 2. Experiments

MWCNTs produced by CVD with a diameter of 10–30 nm and a length of 6–15 μm were used in our experiments. The MWCNTs were treated in a mixture of sulfuric acid and nitric acid (3:1 H_2_SO_4_:HNO_3_) under reflux at 140 °C for 30 min, followed by filtration of the acid mixture. The residue was re-suspended in de-ionized water. This process was repeated several times until the pH of the solution reached neutral. The acid treatment can greatly enhance the solubility of MWCNTs by introducing carboxylic (-COOH) functional groups to the sidewalls and ends of MWCNTs [10]. Next, the MWCNTs were dispersed into pure ethanol at a concentration of approximately 2 μg/mL by ultrasonic agitation for about 1 hour.

**Figure 1.**
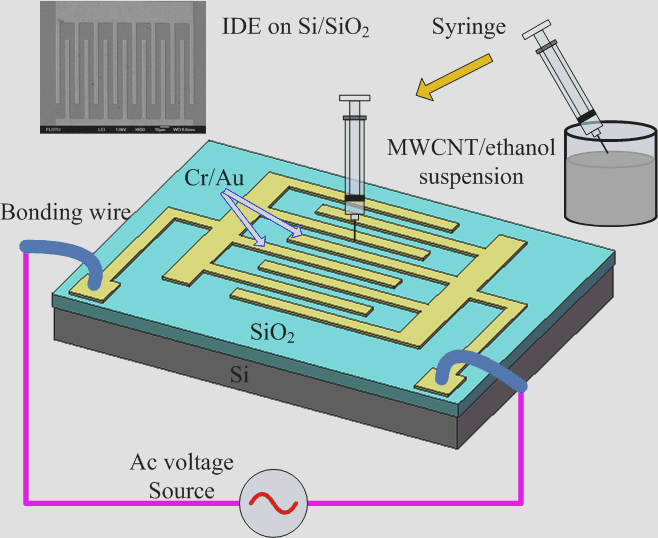
Schematic diagram of MWCNT deposition on IDEs by ac dielectrophoresis.

Figure 1 shows the schematic diagram of MWCNT deposition on IDEs by ac dielectrophoresis. Chromium (Cr, 40 nm) and gold (Au, 300 nm) layers were deposited by sputtering, and patterned by photolithography and etching to form IDEs on a silicon wafer with a layer of SiO_2_ 200 nm thick on Si. The fingers of the IDEs are 120 μm long and 8 μm wide with gaps 8 μm in space. These IDEs have been electrically contacted to a printed circuit board (PCB) by wire-bonding with the DEP.

Approximate 50 μl of the MWCNTs/ethanol solution was dropped to the substrate between IDEs by a syringe. An ac voltage of 20 V (peak to peak) at a frequency of 2 MHz was applied to align MWCNTs on IDEs. MWCNTs dispersed in ethanol were trapped and aligned between IDEs under a positive DEP force. After a desired DEP period of time, the ethanol was evaporated at room temperature.

After DEP and ethanol evaporation, MWCNT networks on IDEs were put into a furnace for repeated thermal annealing. The furnace was heated up to 80°C from room temperature within 10 minutes, kept at 80°C for 30 min, and the furnace was cooling down naturally. The resistance of a MWCNT network at room temperature was measured after each annealing cycle. The electrical characteristics of the networks were measured with a digital multimeter (Keithley SMU 237). Humidity testing experiments were conducted by exposing the MWCNT networks to different relative humidity using a High-Low Temp. & Humi. Chamber (PHC1002-M II, Wuxi Partner Science & Technology Co., Ltd., P.R. China). The RH ranged from 25 % to 95 % at room temperature.

## 3. Results and Discussion

**Figure 2.**
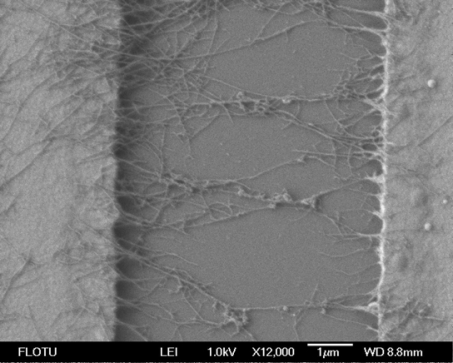
SEM image of a MWCNT network between interdigitated electrodes.

MWCNTs were attracted toward the Au electrodes, and connected across the electrodes, as shown in Figure 2. The density of MWCNTs on IDEs can be controlled by the DEP parameters (e.g. frequency and amplitude), the concentration and volume of MWCNTs.

**Figure 3.**
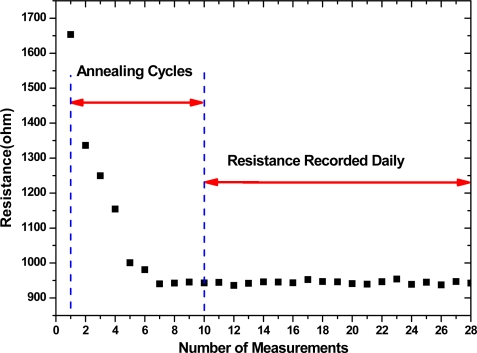
Room-temperature resistance of a MWCNT network during ten repeated annealing cycles and the resistance recorded daily after annealing.

Figure 3 shows the resistance of a MWCNT network measured at room temperature after each annealing cycle, and recorded daily after annealing. The resistance decreased about 45% after eight annealing cycles. The resistance became very stable with a change of less than 1% after eight cycles. The possible reasons for the resistance decrease are that during the thermal annealing processes, the ethanol desorption decreases the CNT resistance and that the contact gaps of the CNT inter-tube junctions and the gaps between CNTs and electrodes are minimized to achieve more direct contact under surface tension in the process of the desorption of ethanol, causing the decrease of the contact resistance.

**Figure 4.**
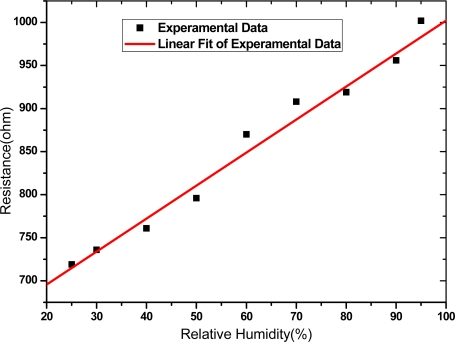
Resistance-relative humidity data and linear fit of a MWCNT network obtained at room temperature.

The resistance of the MWCNT networks was measured at different relative humidity (RH) at room temperature. The resistance increases linearly with increasing RH from 25 % to 95 %, as shown in Figure 4. The sensitivity, S, of a MWCNT network to humidity can be defined as:

(1) $$S=\frac{1}{R_{0}}\times \frac{\Delta R}{\Delta \mathrm{RH}}\times 100\%$$

where ΔR is the change of resistance of a MWCNT network, R_0_ is the resistance measured at RH=25 %, and ΔRH is the change of RH. A sensitivity of 0.5%/%RH was obtained from the slope of the fitted line in Figure 4 and the correlation coefficient of 0.9893 indicates the MWCNT network having a good linearity of resistance response to RH. This result on the resistance of MWCNTs increasing with the RH is consistent with the results on SWCNTs obtained by theoretical calculations [11]. The humidity sensing mechanism of the MWCNT network could be described as follows. MWCNTs usually exhibit a hole transport as like p-type semiconductor for sensing gas/vapor [12]. Water molecules adsorbing on surfaces of MWCNTs transfer electrons to MWCNTs because of an electrical potential difference between the two materials. The electron transfer depletes the concentration of holes in MWCNTs, resulting in an increase of resistance. The amount of water molecules available to be adsorbed by MWCNTs depends on the RH level of the environment. The higher the RH level, the more water molecules are adsorbed and more electrons are transferred, causing the increase of resistance.

**Figure 5.**
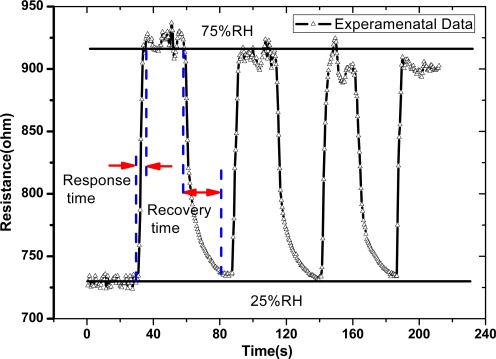
Time response and recovery curve of the MWCNT network from RH=25 % to 75 %.

The response and recovery time of MWCNT networks were tested through adsorption-desorption dynamic cycles, with RH being interchanged between 25% and 75% repeatedly. The response-time curves of several typical dynamic cycles were illustrated in Figure 5. It is estimated that the response time is about 3 s, and the recovery time is about 25 s. Here the response time is shorter than the recovery time, which is the same as the general phenomenon of humidity sensors. The MWCNT network has a good reversibility, although its sensitivity decreases about 7% after four cycles of humidity switch between 25% and 75% RH. This result indicates that the interaction between water vapor and MWCNT networks is mainly dominated by physisorption with a weak bond.

**Figure 6.**
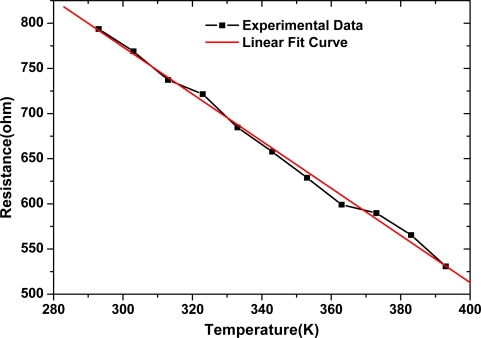
Temperature dependence of the resistance of the MWCNT network.

To determine the effect of temperature on resistances of MWCNT networks, the resistance of the MWCNT network was measured at a temperature range from 293K to 393K. The resistance linearly decreased with increasing temperatures, as shown in Figure 6. The temperature coefficient of resistance (TCR) of the MWCNT network is about −0.33%/K. Hence, the temperature effect on humidity sensitivity of MWCNT networks cannot be neglected, and a temperature compensation is necessary when a MWCNT network is used as a humidity sensor.

## 4. Conclusions

MWCNT networks were deposited on IDEs by ac DEP for humidity sensing. The resistance stability of MWCNT networks was effectively improved through thermal annealing. The resistance of MWCNT network increases with the increase of relative humidity at room temperature. The experimental results show that the MWCNT networks are a promising humidity sensing material with a good reversibility, a high sensitivity of 0.5%/% RH, a good linearity from 25 % to 95 % RH, and short response and recovery times of about 3 and 25 s, respectively. The temperature effect of MWCNT networks on resistance changes cannot be neglected, since MWCNT networks have a negative temperature coefficient of resistance.
